# Gastrointestinal microbiota-directed nutritional and therapeutic interventions for inflammatory bowel disease: opportunities and challenges

**DOI:** 10.1093/gastro/goae033

**Published:** 2024-04-27

**Authors:** Devendra Paudel, Divek V T Nair, Grace Joseph, Rita Castro, Amit K Tiwari, Vishal Singh

**Affiliations:** Department of Nutritional Sciences, The Pennsylvania State University, University Park, PA, USA; Department of Nutritional Sciences, The Pennsylvania State University, University Park, PA, USA; Department of Nutritional Sciences, The Pennsylvania State University, University Park, PA, USA; Department of Nutritional Sciences, The Pennsylvania State University, University Park, PA, USA; Department of Pharmaceutical Sciences and Medicines, Faculty of Pharmacy, Universidade de Lisboa, Lisboa, Portugal; College of Pharmacy, University of Arkansas for Medical Sciences, Little Rock, AR, USA; Department of Nutritional Sciences, The Pennsylvania State University, University Park, PA, USA

**Keywords:** dietary fiber, dysbiosis, fecal microbiota transplant, microbiota restoration therapy, intestinal inflammation, microbial metabolism

## Abstract

Evidence-based research has confirmed the role of gastrointestinal microbiota in regulating intestinal inflammation. These data have generated interest in developing microbiota-based therapies for the prevention and management of inflammatory bowel disease (IBD). Despite in-depth understanding of the etiology of IBD, it currently lacks a cure and requires ongoing management. Accumulating data suggest that an aberrant gastrointestinal microbiome, often referred to as dysbiosis, is a significant environmental instigator of IBD. Novel microbiome-targeted interventions including prebiotics, probiotics, fecal microbiota transplant, and small molecule microbiome modulators are being evaluated as therapeutic interventions to attenuate intestinal inflammation by restoring a healthy microbiota composition and function. In this review, the effectiveness and challenges of microbiome-centered interventions that have the potential to alleviate intestinal inflammation and improve clinical outcomes of IBD are explored.

## Introduction

**Figure 1.**
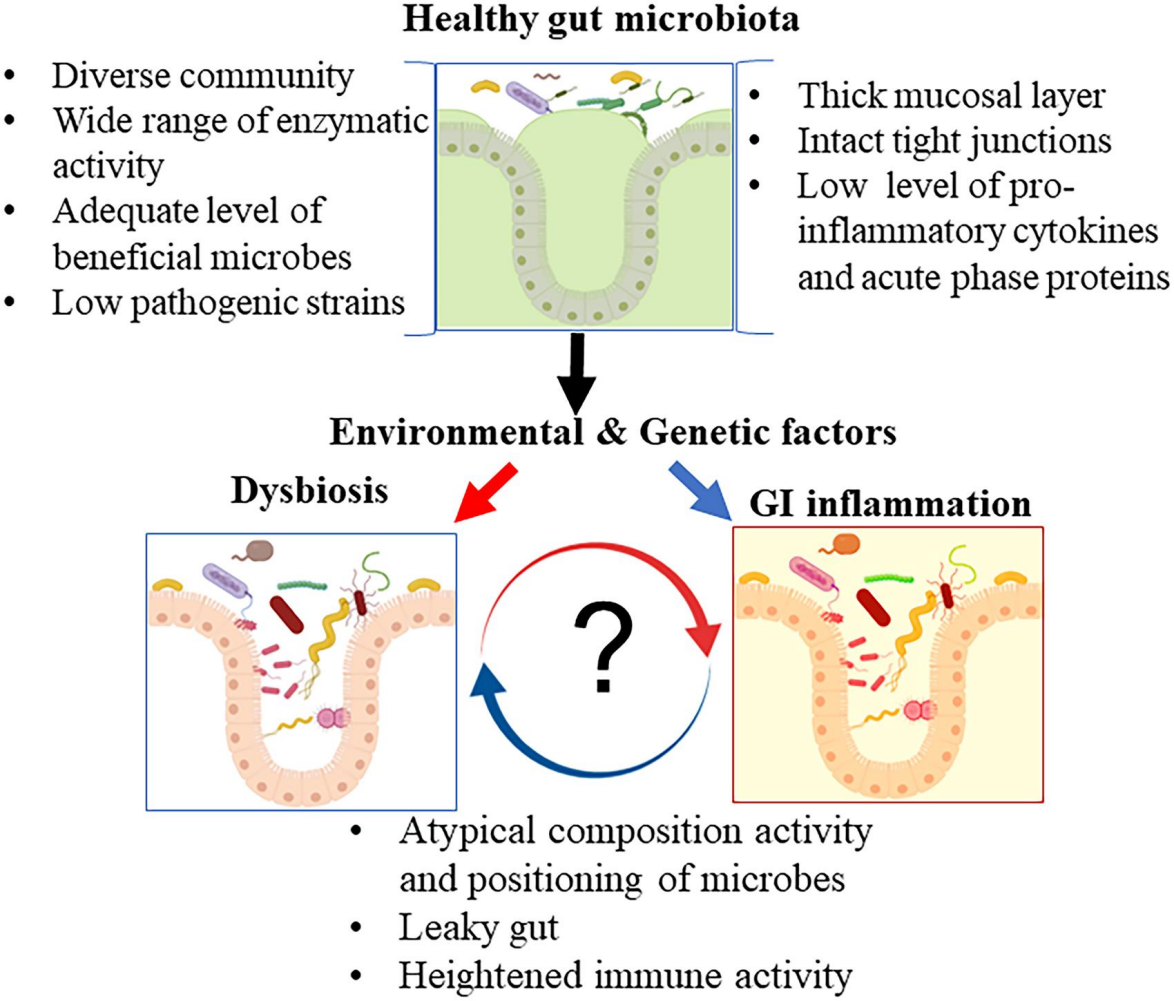
A schematic diagram showing that atypical gut microbiota (i.e. dysbiosis—a relative difference in both composition and metabolic activity of gut microbiota) could be a cause or consequence—or both—of GI inflammation. The right arrow represents GI inflammation that induces dysbiosis. The left arrow represents dysbiosis that elicits GI inflammation. The figure was illustrated with the help of BioRender software (accessed in 2021). GI = gastrointestinal.

Inflammatory bowel disease (IBD) is a chronic inflammatory condition of the gastrointestinal (GI) tract primarily resulting from a dysregulated host immune response to environmental factors, including atypical GI microbiota [1, 2]. Crohn's disease (CD) and ulcerative colitis (UC) are the two most common types of IBD. Patients with CD exhibit discontinuous (patchy), transmural inflammation, affecting multiple layers of the intestinal wall. In contrast, UC is characterized by continuous inflammation, typically limited to the large intestine (colon and rectum). Additionally, inflammation in UC is confined to the inner mucosal lining. Both CD and UC patients tend to have a GI microbiota that is less diverse [3, 4] and exhibit reduced metabolic capacity [5, 6]. A decrease in the number of *Faecalibacterium* is considered to be the “hallmark” of IBD [3], while an increase in *Proteobacteria* and *Actinobacteria* is a characteristic of dysbiosis [7, 8]. Such alterations in the gut microbiome composition can impact microbial metabolome leading to the disruption of intestinal immune function and barrier integrity. However, whether such dysbiosis is an outcome of existing inflammation or has a causative role in IBD development is largely unknown [9] (Figure 1).

The interplay among commensal microbes, the intestinal epithelium, and resident immune cells shapes the composition and function of the gut microbiota and the intestinal immune response [2]. A diet consistently high in ultra-processed, low-fiber foods may disrupt the delicate communication among gut bacteria, the intestinal lining, and immune cells, potentially leading to chronic inflammation, including IBD [10]. Dietary fibers present in fruits and vegetables play a constructive role in preserving the diversity of microbes within the intestines of individuals in good health [11]. Gut microbiota uses these dietary fibers as metabolic substrate and produces short-chain fatty acids (SCFAs) such as acetate, propionate, and butyrate through fermentation. These SCFAs play a pivotal role in governing the intestinal immune response through their interactions with immune pathways [12, 13]. Besides fiber fermentation metabolites, host molecules including bile acids also play a role in host immunity and microbiota composition. Dysregulation of microbial bile acid metabolism is known to contribute to IBD development [14] and the growth of *Clostridioides difficile*—an opportunistic pathogen that infects the large intestine [15]. The role of the SCFAs and bile acids in regulating intestinal immune response through alterations to the gut microbiota will be discussed further within this review.

The role of the dysbiotic gut microbiome in contributing to the adverse immune response found in IBD has led to interest in strategies that either favorably alter the microbiome composition and/or restore the balanced gut microbiome. Prebiotics, probiotics, parabiotics, postbiotics, and synbiotics (Box 1) in dietary components or supplements have been studied for their potential benefit to individuals with IBD and will be discussed in the later parts of this article.

The present therapeutic strategies for the treatment of IBD are focused primarily on managing symptoms rather than a curative or preventive approach [16, 17]. IBD patients are typically treated with 5-aminosalicylate, corticosteroids, antibiotics, immunomodulators, and biologics [16]. Novel biological therapies, including monoclonal antibodies for tumor necrosis factor (TNF), are promising; however, a subgroup of IBD patients who are non-responsive, develop adverse reactions, and/or are at risk of developing secondary infections is further complicating the management of IBD [18]. Herein, there is a need to advance innovative treatment modalities characterized by diminished adverse effects and enhanced clinical efficacy, with the goal of more effectively managing IBD. Preclinical and clinical studies suggest that an imbalanced GI microbiota disrupts the intestinal immune balance, contributing to the development of IBD over time [19, 20]. Therefore, ameliorating the pre-existing dysbiosis by dietary modification or restoring microbiota homeostasis could represent potential approaches. To understand the opportunities and limitations of using nutritional and therapeutic interventions to target microbiota for managing and potentially treating IBD, we conducted a review of relevant research published online. We searched databases such as PubMed, Web of Science, and Google Scholar, focusing on research articles, systematic reviews, and meta-analyses published after 2000. Our search included keywords such as IBD, UC, CD, nutrition, dietary fibers, gut microbiome, and microbiome-based therapeutic approaches for IBD. Precisely, this review delves into previous research that considers the relative contribution of gut microbiota dysbiosis to the onset and exacerbation of IBD. It then navigates through various nutritional interventions, including prebiotics and probiotics, aimed at restoring microbial homeostasis and ameliorating inflammation in IBD patients. Furthermore, the review explores new therapeutic approaches, such as microbiota-restoration therapy and microbiota-targeted small molecules that beneficially modify host–microbe interactions, holding promise for reshaping IBD treatment approaches. Altogether, this review discusses ongoing microbiome-focused investigational therapies, their limitations, emerging opportunities, and challenges associated with developing microbiome-informed interventions for improving IBD.

## Microbiota dysbiosis elicits the disease or disease induces dysbiosis: the causation conundrum in IBD

Several studies suggest that a dysbiotic GI microbiome plays a role in the development of IBD [21, 22]. However, whether an alteration in the composition and metabolic activity of the GI microbiota in IBD patients constitutes a cause or an outcome of the inflamed environment within the intestines remains to be established. The high variability between studies and the lack of a common microbe identified across all studies limit our ability to pinpoint a specific group of microbes that drives or dampens IBD pathogenesis. In a healthy host, the diverse microbial composition plays a symbiotic role in the digestion of dietary fibers, synthesis of specific vitamins, and a favorable modulation of intestinal immune responses [23]. Moreover, a diverse microbiome prevents the colonization of pathogenic bacteria. Environmental influences, such as prolonged usage of broad-spectrum antibiotics or a deficiency in fiber within the diet, disrupt symbiotic rapport between the host and the microbiota. Although it remains to be determined whether dysbiosis directly causes inflammation in IBD patients, preclinical studies using germ-free mice colonized with specific bacteria have identified strains capable of triggering intestinal inflammation. For instance, germ-free mice lacking interleukin-10 (IL-10 KO) developed mild inflammation in the cecum, while *Enterococcus faecalis* colonization led to distal colitis [24]. Interestingly, colonization with *Candida albicans*, *Lactobacillus casei*, *L. reuteri*, *L. acidophilus*, a *Bifidobacterium* sp., *Lactococcus lactis*, or a *Bacillus* sp. did not induce inflammation in any part of the GI tract of germ-free IL-10 KO mice [24]. These findings in mice suggest that IBD could result from the presence of specific bacterial species, although this remains to be verified in patients with IBD. A strong correlation exists between dysbiosis and IBD, though a causal relationship between specific bacterial species and inflammation remains unclear (Figure 1). Additional research is required to understand the complex relationship between genetics, GI microbial composition, and inflammation in the pathogenesis of IBD.

## Limitations of present IBD therapies

Therapeutic strategies for IBD chiefly aim to manage the disease by reducing ongoing intestinal inflammation and preventing sustained inflammation-induced tissue damage and fibrosis. Principal anti-inflammatory and immunosuppressive drugs currently in use to manage IBD are 5-aminosalicylate (5-ASA), corticosteroids (such as glucocorticoids [GCs]), immunomodulators (e.g. methotrexate), and biologics (e.g. TNF antagonists) [25]. The main goals of these drugs are to achieve and maintain a state of remission, prevent hospitalization, and improve the quality of life (QOL) to the best possible extent. For a subset of IBD patients with reduced response to potent agents such as biologics, dose escalation and increased frequency become necessary.

Corticosteroid therapy is an effective treatment in reducing disease activity and induction of remission in patients with IBD. However, the chronic use of GCs can produce central nervous system, musculoskeletal, metabolic, dermatologic, cardiovascular, and immunological adverse effects [26]. In addition, GCs have adverse GI effects such as indigestion and bleeding in the upper abdomen upon eating, which can exacerbate IBD [27]. Second-generation GCs, such as budesonide and beclomethasone dipropionate, primarily applied topically, represent a better alternative for systemic GCs. 5-ASA, a standard therapy used for mild to moderate UC, can produce acute and chronic adverse effects such as nausea, abdominal pain, and diarrhea [27]. Biological therapies that include TNF-α inhibitors, such as infliximab, adalimumab, golimumab, and certolizumab pegol, are being effectively used to suppress excessive intestinal inflammation for inducing and maintaining remission in patients with IBD [28]. However, treatment failures associated with anti-TNF therapy, including primary non-responsiveness to treatment or secondary loss of response to these drugs, can occur due to immune-mediated neutralizing antibodies [28–30]. The use of these biological therapies is associated with a higher incidence of specific types of opportunistic infections [8, 31], which underscores the crucial need for a new line of therapeutic agents and adjuvant therapies that deliver sustained remission and improve clinical outcomes of IBD. Beyond conventional treatments, alternative therapeutic approaches such as microbiome-targeted therapy hold promise in managing IBD symptoms and improving the QOL for IBD patients.

## The effect of prebiotics, probiotics, and synbiotics in IBD

**Figure 2.**
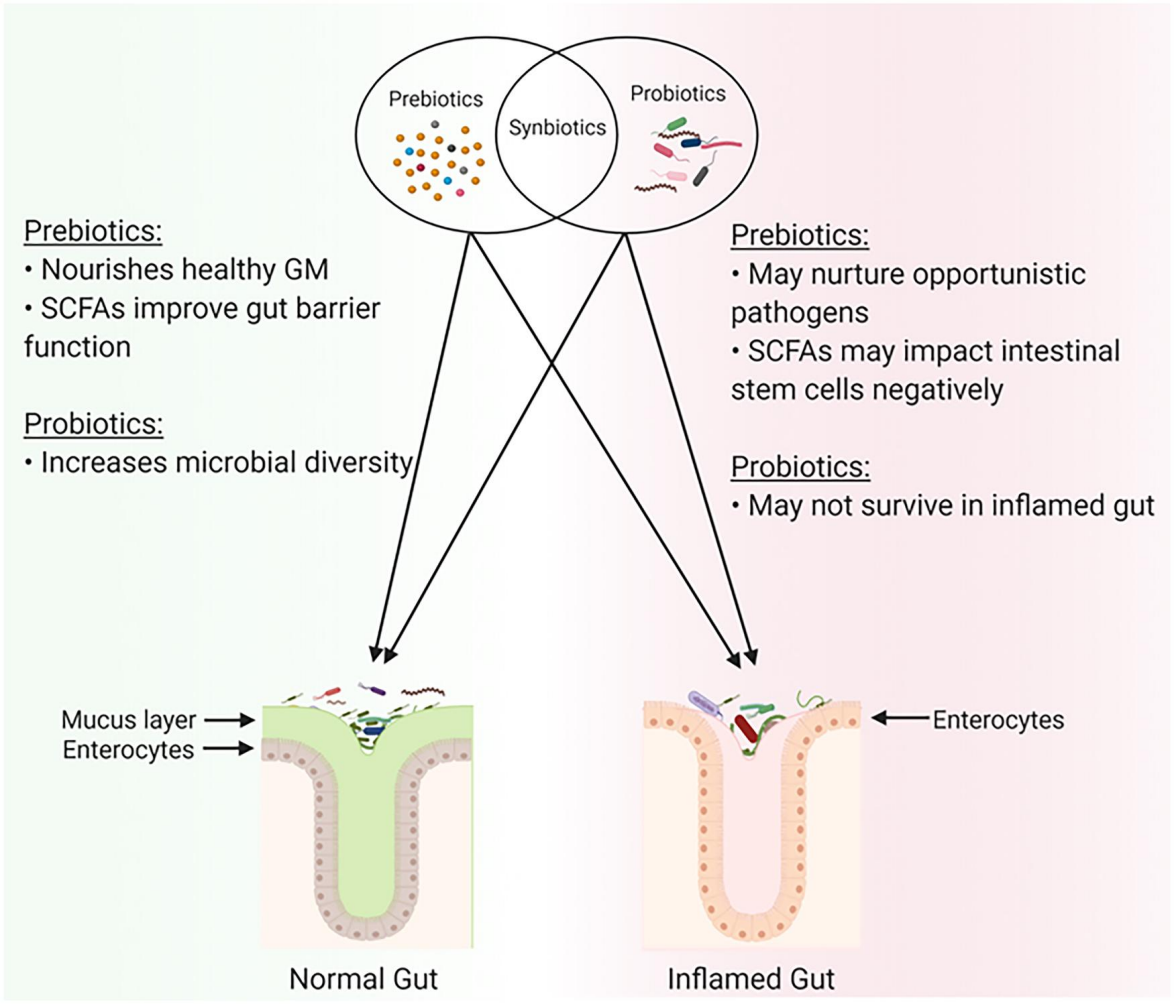
A schematic diagram showing the effect of prebiotics, probiotics, and synbiotics. In healthy individuals, the consumption of prebiotics, probiotics, and synbiotics has been reported to be beneficial in decreasing inflammation and protecting the GI tract from colonization by opportunistic pathogens. However, in IBD, the effect of consuming prebiotics, probiotics, and synbiotics may be detrimental and could further exacerbate inflammation, nurture opportunistic pathogens, and negatively impact intestinal progenitor cells. The figure was illustrated with the help of BioRender software (accessed in 2021). IBD = inflammatory bowel disease, GI = gastrointestinal, SCFAs = short-chain fatty acids.

Microbiota-accessible carbohydrates such as fermentable dietary fibers (FDFs) play pivotal role in maintaining a symbiotic relationship between host and microbiota in the GI tract. Prebiotics are types of FDFs that selectively nourish beneficial gut bacteria. A reduced intake of naturally occurring FDFs favors the expansion of colonic mucus-degrading bacteria that compromises the GI barrier function and elicits colonic inflammation [32–34]. Bioactive products, such as amines, phenols, and sulfur compounds, derived from microbial protein fermentation, also increase the likelihood of intestinal inflammation [35]. In a seminal study [33], the authors observed that fiber deprivation significantly increases the growth of mucus-eroding microbiota and susceptibility to the intestinal pathogen *Citrobacter rodentium*. Supporting the notion that FDFs are beneficial for GI health, an intervention with psyllium fiber successfully attenuated dextran sulfate sodium (DSS)-induced experimental colitis [36]. Recently, Bretin *et al.* demonstrated that psyllium fiber elevates bile acids and engages the farnesoid X receptor to provide protection against colitis [37]. These empirical observations underscore the critical role of dietary fiber in regulating intestinal inflammation. While naturally occurring FDFs are known to benefit gut microbiota and intestinal health, supplementation with highly processed fibers has been shown to exacerbate colitis in preclinical models [38–41]. These detrimental effects, particularly of inulin-type fructans, have also been observed in a subgroup of IBD patients [42]. Along the same lines, research studies are exploring the efficacy of probiotics, live beneficial bacteria, in attenuating intestinal inflammation; alleviating common IBD symptoms such as diarrhea, abdominal pain, and bloating; and ultimately improving the QOL of IBD patients. Synbiotics, which consist of a mixture of both prebiotics and probiotics, are becoming more popular to aid in the survival and growth of beneficial bacteria in the lower GI tract [43, 44]. Overall, dietary intervention(s) that can support (i.e. prebiotics), restore (ie probiotics), or both (i.e. synbiotics) may represent a viable strategy to maintain a healthy GI tract and mitigate intestinal inflammation [44] (Figure 2).

### Prebiotics in IBD prevention: potentials and limitations

According to the International Scientific Association for Probiotics and Prebiotics (ISAPP), a prebiotic is “a substrate that is selectively utilized by host microorganisms conferring a health benefit” [45]. Specifically, prebiotics are classified as a substrate that must be resistant to the conditions of the GI tract, fermentable by the GI microbiota, and cannot be hydrolysed by the enzymes of the small intestine. Prebiotic consumption has been considered safe for healthy adults, demonstrating the ability to enhance microbial diversity and promote health. Nonetheless, it remained elusive whether prebiotics yield similar benefits for individuals with IBD [42].

The effects of prebiotics on colonic inflammation have been evaluated in experimental models of IBD. Fructan (inulin and fructooligosaccharides [FOS]), which is a linear chain of fructose with a β (2→1) linkage, and galacto-oligosaccharides (GOS), which is a chain of lactose molecules connected by β (1→6), β (1→3), and β (1→4) linkages [46], are among the most-evaluated prebiotics regarding their effects on intestinal inflammation. The daily co-administration of inulin and FOS increases the proliferation of *Lactobacillus* and *Bifidobacteria*, decreases mucosal pro-inflammatory cytokines IL-1β and IFN-γ, and improves crypt damage in dextran DSS-fed HLA-B27 transgenic rats [47]. In a mouse model of DSS-induced UC, administration of low- and high-molecular-weight oat-derived beta-glucan decreased myeloperoxidase activity, malondialdehyde levels, and nitric oxide levels [48]. Similarly, intervention with purified pectin with various degrees of esterification significantly attenuated intestinal epithelial injury, inflammation, and oxidative stress in an experimental model of colitis [49]. Moreover, studies using germinated barley [50], psyllium fiber [37], and xylooligosaccharides [51], which are not primarily considered as prebiotic fibers, exhibited a protective response to intestinal inflammation.

Although significant evidence suggests that dietary fibers and their bacterial fermentation products benefit the management of IBD, it is crucial to consider these findings in the context of both healthy and diseased (ongoing intestinal inflammation) states. Emerging data suggest that FDFs such as inulin may worsen symptoms in some patients with IBD [38, 39, 42]. This highlights the need for further research on the specific benefits of different fiber types in IBD. Our own work has revealed that the dietary fiber inulin exacerbates intestinal inflammation [38] and colitis-associated colon tumorigenesis [39], while structurally distinct fiber pectin offers protection against colitis, emphasizing the potential risks and benefits associated with distinct fiber types [38]. This observation finds support in other studies as well [40, 41]. The use of FDFs, such as arabinoxylans, β-glucans, β-fructans, and pectin shows promise in promoting beneficial gut microbes and metabolites in the distal gut. However, extensive research is needed to fully understand their effects in isolation or in combination, especially in the inflamed intestinal environment of IBD patients.

### Probiotics: do they improve IBD?

The ISAPP defines probiotics as “live microorganisms that, when administered in adequate amounts, confer a health benefit on the host.” This definition encompasses only those microbial species proven to provide health advantages through rigorously controlled studies. Any live cultures of traditional fermented foods lacking evidence of efficacy are excluded [52]. Certain strains of *Lactobacillus* and *Bifidobacteria* are the most-used probiotic microbes [53]. A long-term study examining the effects of probiotics in patients with UC reported that patients receiving mesalazine (5-ASA) and a probiotic blend of *L. salivarus*, *L. acidophilus*, and *Bifidobacterium bifidus* had an improvement in their clinical symptoms, based on the Modified Mayo Disease Activity Index and Physician’s Global Assessment, compared with patients receiving mesalazine alone. The combined therapy group had a decrease in stool frequency and clinical improvement over the 24-month study [54]. Similarly, probiotic yogurt containing *L. rhamnosus* and *Limosilactobacillus reuteri* increased the T-reg cell count in IBD patients after 30 days of consumption [55]. In a double-blind, placebo-controlled trial in UC and CD patients, inflammation was decreased in UC but not in CD patients after consuming a multi-strain (*L. rhamnosus*, *L. plantarum*, *L. acidophilus*, *E. faecium*) probiotic [56]. Overall, the available data on the therapeutic efficacy of probiotics for improving clinical symptoms in IBD patients remains limited. It is conceivable that specific factors such as the extent of ongoing intestinal inflammation in IBD patients may influence treatment response. Recently, the utilization of genetically engineered bacteria and postbiotics has introduced a novel avenue for addressing intestinal inflammatory conditions [57–60]. These genetically modified probiotics hold the potential to exert a dual impact: they can help regulate intestinal dysbiosis while simultaneously releasing therapeutic compounds directly into the intestine. This approach circumvents the need for systemic drug administration and mitigates associated systemic side effects. Probiotics show promise in animal studies but their effectiveness in humans may be limited by stomach acids, bile salts, and active inflammation in patients with IBD [61].

### Synbiotics: the promise of combined modulation of the GI microbiota in IBD

Synbiotics are designed to maximize the potential of both probiotics (live bacteria) and FDFs (nourishment for gut bacteria) [62]. A randomized–controlled trial [63] compared the effects of synbiotics, prebiotics, and probiotics on the QOL in patients with UC where participants received either *B. longum* (probiotic), psyllium fiber (an FDF), or a combination of both (synbiotic). The study found the greatest improvement in QOL scores in the synbiotic group [63]. A systematic review and meta-analysis of the use of probiotics, prebiotics, and synbiotics in IBD found that synbiotics were associated with a significant improvement in remission rates of UC [64]. Still, identifying synergistic combinations of prebiotics and probiotics, where prebiotics enhance the benefits of added probiotics, remains a complex challenge. The future of synbiotics relies on a methodical formulation and design process. This involves the careful selection of synbiotics that harmonize and enhance each other's effects, guided by their specific mechanisms of action and the intended health benefits [65].

Despite enormous promise and interest, driven primarily by preclinical studies, there are only a few limited, well-designed, randomized–controlled trials to support the concept that microbiome-targeted nutritional and probiotic approaches may mitigate clinical symptoms and cure IBD. In preclinical studies, it is evident that prebiotics, probiotics, and synbiotics can maintain a diverse and healthy GI microbiota and beneficially modulate intestinal immune activity [37, 66–68]. The efficacy of these treatments suggests that consuming fruits and vegetables containing fermentable fibers increases the QOL in IBD patients. However, large-scale randomized–controlled trials are needed to validate the potential protective efficacy of microbiome-centered dietary interventions.

## Microbiome-targeted investigational therapies for IBD

### Microbiome-based therapeutic approaches

Commensal and symbiotic microbes co-evolved with the host and synthesize certain vitamins (K and B vitamins); biodegrade glycosaminoglycans; and produce SCFAs, secondary bile acids, and essential amino acids [69, 70]. The potential for gut microbiota and its metabolites to regulate intestinal immune homeostasis has fueled interest in therapeutic approaches targeting the restoration of healthy microbial composition and their metabolic activity (Box 2).

### Restoring healthy microbiota

Fecal microbiota transplantation (FMT) involves the therapeutic infusion or engraftment of homogenized fecal suspension from healthy donors into the GI tract of patients suffering from IBD [71]. This approach has gained significant attention because of the successful implementation of FMT in treating *Clostridium difficile* infection [72, 73]. Despite the huge promise of FMT in limiting *C. difficile* infection, standardization among various studies, particularly regarding the route of administration, dose, volume, frequency, and method of preparation (i.e. fresh, frozen, single-dose, or pooled sample) of the fecal transplant, is required to compare the efficacy of various FMT trials appropriately [74, 75]. Analysing the effectiveness of FMT interventions becomes intricate when pooled samples are involved, as attributing outcomes to specific donors within the pool becomes difficult. Moreover, the risk of transferring opportunistic pathogens such as *Escherichia coli* increases with the number of donors in the pool.

Because there are many unknown factors associated with the efficacy and safety of FMT, more defined and targeted microbial populations are being investigated as therapeutic strategies to treat IBD. Microbiota restoration therapy is targeted to ameliorate the dysbiosis due to IBD by using defined microflora [76]. Data suggest that the gut bacteria of individuals with IBD differ in composition and metabolic capacity compared with those of healthy individuals. Relatively lower levels of *Faecalibacterium prausnitzii*, *Blautia faecis*, *Roseburia inulinivorans*, *Ruminococcus torques*, and *C. lavalense* were shown in CD and relapsed CD patients [77–79]. These bacterial genera can produce SCFAs, secrete antimicrobial peptides, produce anti-inflammatory effects, and enhance certain immune responses that could restore perturbed intestinal homeostasis [9, 52, 80]. Currently, several microbiome companies are conducting research trials to evaluate potential of defined microbial populations (SER-287, SER-301, VE202, FIN-524, FIN-525, RBX2660, and MET-2) for the treatment of IBD (Table 1). Recent approval of Vowst by the U.S. Food and Drug Administration (FDA)—the first orally administered microbiota-based therapeutic for preventing recurrent *C. difficile* infection—underscores the potential and promise of microbiota-replacement therapy for improving outcomes in IBD patients.

**Table 1.** Microbiota restoration therapy studies

Therapeutic candidate	Conditions	Composition	Current phase	Mode of action	Clinical trial ID
SER-287	UC	Consists of a complex and diverse bacterial spore ecology	Phase 2b	Decreases immune activation	NCT03759041
(Seres Therapeutics)					
VE202	IBD	A defined consortium of live bacteria designed to modulate the activity of regulatory T cells	Phase 2	Designed to modulate the activity of regulatory T cells	NCT05370885
(Vedanta Biosciences)					
FIN-524	UC	Rationally selected microbiota product, lyophilized bacterial strains grown in pure culture	Preclinical	Immuno-modulatory properties	Not applicable
(Finch Therapeutics)					
FIN-525	CD	Rationally selected microbiota product, lyophilized bacterial strains grown in pure culture	Preclinical	Immuno-modulatory properties	Not applicable
(Finch Therapeutics)					
RBX2660	Pediatric UC	Live microbes from screened human donors	Phase 3	Immuno-modulatory properties	NCT03931941
(Rebiotix)					
MET-2	UC	Defined consortium of human commensal bacteria derived from a healthy donor	Phase 1	Gut microbiome restoration and mucosal healing	NCT02865616
(Mount Sinai Hospital, Ontario)					

IBD = inflammatory bowel disease, UC = ulcerative colitis, CD = Crohn's disease.

### Modifying host–microbe interactions: future therapeutics

Therapeutic strategies can be developed by modifying microbial metabolism or interfering with the host–microbe interactions. These promising therapeutic approaches are elegantly discussed [81] and are tabularized in Table 2. Microbially derived metabolites have been extensively evaluated for their potential use as medicinal drugs, although most of these compounds have not been advanced to clinical trials (Table 2). The compound EB8018 is a FimH inhibitor that specifically blocks the binding of adherent-invasive *E. coli* to the intestinal epithelium [82] and is undergoing evaluation in a clinical trial in CD patients. Peptide SG-2-0776 is a compound purported to bind to the interface of the cell membrane and the extracellular matrix to promote mucosal healing (Second Genome pipeline) [81]. SG-2-0776 is currently being evaluated in a preclinical model for IBD. The compounds SYMB-104 and SYMB-202 have been shown to decrease intestinal inflammation in mice by increasing the levels of T-reg cells [81]. Collectively, microbial metabolites, which maintain immune homeostasis, mucosal integrity, and immune maturation, are being evaluated as potential therapeutics to cure IBD.

**Table 2.** Microbiome-targeted small molecules for IBD

Therapeutic candidate	Conditions	Phase	Mode of action	Company
EB8018	CD	Phase Ib	FimH inhibitor that blocks the adherence of invasive *Escherichia coli* to the intestinal epithelium protein, FimH	Enterome Biosciences
SG-2-0776	IBD	Preclinical	Mechanism of action yet to be established. Hypothesized to bind to the proteins at the interface of the cell membrane and the extracellular matrix, facilitates mucosal healing	Second Genome
SYMB-104 (polysaccharide A)	IBD	Preclinical	The compound is naturally produced by GI commensal organisms and stimulates the production of regulatory T cells to suppress inflammation	Symbiotix Biotherapies
SYMB-202 (outer membrane vesicles)	IBD	Preclinical	Stimulates regulatory T cells, suppresses inflammation	Symbiotix Biotherapies
PEM compounds	UC	Preclinical	Modulates the enteric signaling network (composed of GI immune cells, the enteric nervous system, and the GI microbiome) in a GI region-specific manner to elicit positive outcomes	Kintai Therapeutics

IBD: inflammatory bowel disease; UC: ulcerative colitis; CD: Crohn's disease. Table 2 is adopted from reference [81].

## Future perspectives

Our understanding of the interactions between dietary macronutrients, GI microbiota, and their effect on inflammation and intestinal pathology is rapidly evolving. Considering the complex nature of IBD and the heterogeneity in pathology and symptoms in patients, we are still in the nascent stage of determining the combinations of microbiota-targeted therapy that can attenuate the clinical complications in IBD patients. Emerging data from gut microbiome-targeted therapies, focusing on preventive strategies for IBD, are encouraging. These early studies indicate the potential of microbiome manipulation in managing and potentially preventing this chronic inflammatory condition. However, their translation into clinical use requires a detailed mechanistic understanding of the enzymatic activities of microbial population and their virulence, particularly in the inflammatory environment. Moreover, the effectiveness of these treatments hinges on establishing whether dysbiosis triggers and fuels intestinal inflammation in this specific subset of IBD patients. Eventually, large-scale randomized–controlled trials are needed to optimize the response of microbiome-directed treatments in IBD patients to alleviate intestinal inflammation and manage IBD effectively.

## Authors’ contributions

D.P. and V.S. conceived and conceptualize the review. D.P., D.VT.N., G.J., and S.V. prepared the original draft. D.P. and D.V.T.N. completed graphics and visualization. D.P., D.V.T.N., G.J., R.C., A.K.T., and V.S. reviewed and finalized the draft. All authors read and approved the final manuscript.
